# An Umbrella Review of Systematic Reviews on Food Choice and Nutrition Published between 2017 and-2019

**DOI:** 10.3390/nu11102398

**Published:** 2019-10-07

**Authors:** Federico J. A. Perez-Cueto

**Affiliations:** Department of Food Science, Section for Food Design and Consumer Behaviour, University of Copenhagen, Rolighedsvej 26, 1958 Frederiksberg, Denmark; apce@food.ku.dk

**Keywords:** systematic review, food choice, nutrition, consumer behaviour, healthy eating

## Abstract

The objective of this umbrella review was to provide an update on the latest knowledge in the field of food choice and nutrition. Databases Scopus and ISI-Web of Science were searched for “food choice” AND nutrition. Papers were included if they were systematic reviews published between January 2017 and August 2019 on any subpopulation group. In total, 26 systematic reviews were kept. Data were extracted with a predetermined grid including first author, publication year, country, population group, explanatory constructs (intervention focus) and reported outcomes. Common indicators for outcome measures on food choice and nutrition studies are nutrition knowledge, healthy food choices, food purchases and food and nutrient intake. The most common strategy implemented to alter food choice with a nutritional aim is nutrition education, followed by provision of information through labels. Among children, parent modelling is key to achieving healthy food choices. In general, combining strategies seems to be the most effective way to achieve healthier food consumption and to maintain good nutrition in all age groups.

## 1. Introduction

The current food system is facing serious challenges and envisages certain disruption in the coming years, particularly in the context of climate change [1]. The year 2019 began with the call by the EAT-Lancet commission for the world to change food consumption in order to remain within planetary boundaries [2]. They specifically called for a population change towards diets mainly of plant origin and using minimally processed foods. This statement parallels most of the national and international recommendations about healthy and sustainable diets [3,4]. Achieving such targets requires immediate action from all societal actors, and it requires a change in food habits. Unfortunately, although societies have been systematically investing on the promotion of healthy food consumption [5,6,7], the population at large is still under-consuming foods of plant origin (fruits, vegetables, pulses and nuts) [8,9,10], while overconsuming foods of animal origin (meat, dairy, eggs poultry) and highly processed foods (rich in added sugars, added salt, and in added lipids, mostly saturated fats or trans-fats) [11]. Therefore, it seems that many of the efforts made by large national campaigns to promote healthier eating have not been successful in achieving healthier food consumption patterns, or in reducing malnutrition, in particular obesity [5,6] which can be prevented through a plant-based diet rich in fresh and healthy plant foods [12].

The fact that food choices affect nutritional status [12,13] is common sense. Consumers at large have been driving the rise in the demand for healthy, sustainable and ethical food products [14]. Food choices are of interest to several actors in the food system, as they drive, e.g., the demand for industrial production [15], or the demand for provision of public health care, as unhealthy dietary choices result from a pervasive obesogenic environment where consumers are manipulated by clever marketing strategies [16].

It is in the context of such knowledge that this paper aimed to providing an update on the latest knowledge in the multi-disciplinary field of food choice and nutrition through a systematic review of systematic reviews, and to serve as a framing paper for *Nutrients*’ Special Issue on Food Choice and Nutrition. The additional aims were to identify the larger fields of research where interactions are possible, and the outcome indicators related to food choice and nutrition.

## 2. Methods

A systematic review of reviews was performed following the Preferred Reporting Items for Systematic Reviews and Meta-analyses (PRISMA) guidelines [17] in the two major English language databases Scopus and ISI Web of Science (WoS). The search strategy had the following syntax in Scopus: TITLE-ABS-KEY (“food choice” AND nutrition) AND (LIMIT-TO (PUBYEAR, 2019) OR LIMIT-TO (PUBYEAR, 2018) OR LIMIT-TO (PUBYEAR, 2017)) AND (LIMIT-TO (DOCTYPE, “re”)). The search strategy performed in Web of Science was as follows: TOPIC: (“food choice” AND nutrition) Refined by: DOCUMENT TYPES: (REVIEW) AND PUBLICATION YEARS: (2019 OR 2018 OR 2017) Timespan: All years. Indexes: SCI-EXPANDED, SSCI, A&HCI, CPCI-S, CPCI-SSH, BKCI-S, BKCI-SSH, ESCI, CCR-EXPANDED, IC.

The inclusion criteria were defined using the following PICOS-tool (Population, Intervention, Comparison, Outcome, Study design): Population to be considered—all age groups. Interventions could be behavioural (e.g., nudges, social media), provision of information (e.g., nutrition education, labels), nutritional (counselling, cooking skills). Outcomes should incorporate nutritional status, food choices, food consumption or purchase, food or nutrient intake, or consumer relevant data (e.g., knowledge, attitudes). Comparison between exposed vs. non-exposed or when participants were their own controls. Study design: Inclusion of Systematic Reviews on food choice and nutrition conducted following the PRISMA guidelines [17,18] or the Joanna Briggs Manual if stated as Scoping Review [19]. Reviews that did not report themselves as systematic were checked thoroughly as some “scoping reviews” or “umbrella reviews” apply systematic review methodology, and thus, were eligible. Narrative reviews were omitted. Data were extracted with a predetermined grid (Table 1) including the name of the first author, the year of publication, the country where the review was performed, the population group, the explanatory constructs (determinants, intervention) and the reported outcomes.

**Figure 1:**
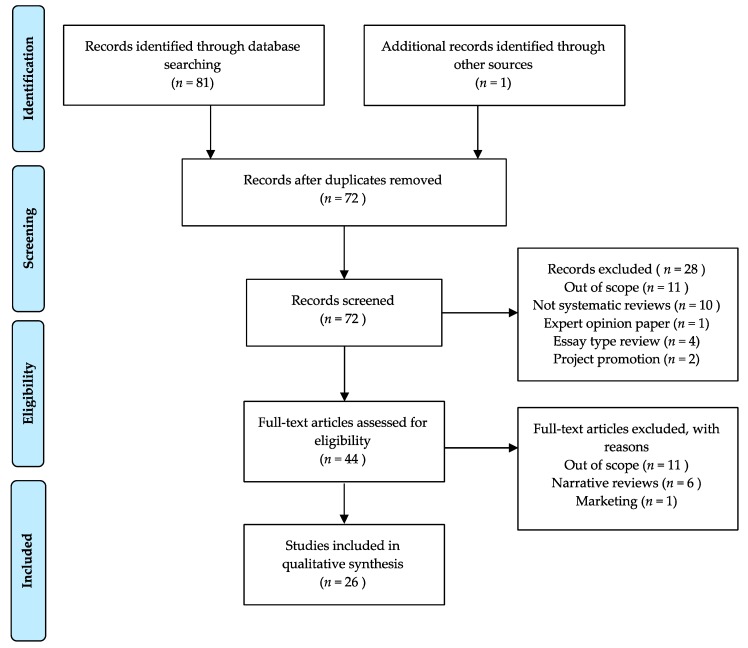
PRISMA flow-diagram for the selection of studies.

Initially, 59 items were retrieved from Scopus (21 retained: 26 out of scope papers title and abstract screening; 12 not meeting inclusion criteria) and 22 items from WoS (10 duplicates, 5 out of the scope and 4 retained). One paper was added by the author. Figure 1 shows the PRISMA flow-diagram describing the process to retain 25 reviews.

No further ethical considerations were made as this is a systematic review of the literature and thus, it did not deal with individual data or with sensitive information.

## 3. Results

Table 1 shows the characteristics and main outcomes of the papers revised. A narrative synthesis approach was chosen, as the papers revealed a large heterogeneity with results that are not comparable in the same units. This narrative synthesis focuses on the main lessons from this review with a focus on the research fields where “food choice and nutrition” papers are of interest, the methods applied, and focusing on the comparability of studies. Table 2 shows a synthesis of interventions to promote healthy eating by age group.

### 3.1. Reviews on Studies about Food Choice and Nutrition in Adult or the General Population

The most common intervention applied among adults or the general population is providing information to promote informed choices as a way of empowering consumers. Provision of information has different expressions: nutrition education [28,32,42], labelling [24,33,38,39,44], nutrition information in supermarkets and menus and packaged foods [38]. However, it also includes improved food literacy [42] as an empowering tool to help consumers make healthier food choices. In all cases the reported effects on behaviour were modest but positive in terms of improved dietary quality [40,41], enhanced food choices and actual food consumption [28,38]. Findings about the effectiveness of labels are contradictory. On the one hand, one previous study suggested that health claims on labels have a moderate influence on choice [33], while on the other hand, another study reported that effects were not significant [44].

Of note, one review reported the effectiveness of nudges [38] in promoting healthier choices that underscore modest and significant effects of interventions, such as altering the placement and properties of food choice (improved food choice) or menu labels helping in the reduction of energy intake and at the same time, improving the quality of the choices.

Liking foods is a determinant of dietary intake and food choice, however, only one review [31] focused on the assessment of such association. Although hedonic measurements were likely to have a link with dietary intake, the level of the association is not consistent, particularly for people who like sweet taste vs. those who dislike it.

### 3.2. Reviews on Studies About Food Choice and Nutrition in Adolescents or Young Adults

Adolescence is a time when food behaviours consolidate and have the tendency to track into adulthood [46]. About one third of the retained studies focused on adolescents or young adults. One review [23] reported on the use of social media as a complementary component of larger nutrition interventions and highlighted its potential benefit for short-term changes in behaviour. Hsu et al. [25] underscored behaviour change techniques among adolescents including social media. Interestingly, improvements in two key healthy eating behaviours, namely increased fruit and vegetable intake and reduction in sugar sweetened beverages, were achieved after the application of techniques such as goal setting, getting social support or demonstration activities. Adolescents welcome initiatives to help them make healthier choices, according to some of the reviews reported by Bauer and Reitsch [38].

Menu labelling could be effective in helping children and adolescents in reducing energy purchased and energy intake as demonstrated in artificial/lab settings, however, findings from controlled settings are difficult to replicate in real-life scenarios [45]. Contextual labels (e.g., traffic lights) seem to be more efficient in supporting healthier choices [24].

Powell [30] suggested that healthy foods should be made as convenient, practical, and interesting as possible for young consumers; interesting foods (designed by FF chains & promoted as specials) give a sense of adventure for young males but leaves them vulnerable to unhealthy or unsustainable food choices. Social support, whether from peers or family members, can have both positive or adverse effects on dietary behaviours and eventual onset of overweight/obesity. Family members and sharing meals with the larger family was associated with better eating habits in early adolescence, but not in older ages [26,47]. Particularly, the presence of grandparents at home might contribute to a healthier weight status among mobile/migrated families but not in societies with little geographic mobility [22].

### 3.3. Reviews on Studies about Food Choice and Nutrition in Children (Infants and or School Age Children)

The two studies by Hosseini-Esfahani [40,41] supported the principle of promoting plant-based diets through different life stages, and that DASH [48] is an adequate quality index for diet quality evaluation. Adherence to DASH was associated with a reduced chance of chronic disease both in adults and children. Strategies for coping with healthy recommendations towards reduced salt consumption comprised exercising self-control at the family’s meal table, replacement of salt with spices, and reduction or avoidance of highly processed foods [39].

The review by Matwiejczyk et al. [21] advocated for combining strategies of nutrition education, changes in the choice environment and in combination with adequate policies to achieve healthier nutrition in children. It also highlighted the importance of role models for children (parents, educators) to achieve success. In Germany, a significant reduction of sugar sweetened beverages consumption by pre-school and school children was the successful result of implementing adequate policies [20], particularly the ban of advertisements promoting unhealthy foods. The principal recommendations, echoing those for healthy and sustainable diets, remain to eat plenty of foods of plant origin, to drink mainly water, to reduce foods of animal origin (milk, dairy, meat, fish, eggs), and low or no consumption of added sugar and sweets [12,13,49].

One study addressed women during pregnancy, lactation and post-partum [37] in middle- and low-income countries. It highlighted the role of health practitioners in providing adequate information on healthy dietary habits within the first 1000 days of life, but also the role of the wider society in providing economic support in this vulnerable period in the lives of mothers and children.

### 3.4. Review Addressing Food Choice and Nutrition Among Older Consumers

This systematic review [36] concluded that the most effective way to improve the quality of older consumers’ diets is by a comprehensive approach to a healthier lifestyle that includes nutrition education, more physical activity, but also that alters the availability of foods considered to be healthier by most recommendations (e.g., foods of plant origin, such as nuts, fruits, vegetables, pulses, olive oil), and thus improving the quality of the diet. It is important to consider that sensory characteristics [31,36,50] of foods for older people are to be underscored during the design of meals and other food products for this consumer segment.

### 3.5. General Apprisal

By far the most common intervention applied towards improving nutritional status and healthier food consumption is nutrition education in its different forms. This review supports the statement that nutritional knowledge is necessary but not sufficient to achieve substantial and long-term behavioural change. Most of the studies showed that the effect of nutrition education alone is limited if measured as behaviour change (e.g., increased consumption of healthy foods such as fruits and vegetables) [21,28,32,36,37,42]; many of the interventions on labelling [24,33,38,39,44,45], those of setting standards for catering and thus changing the default [20], or investing in the menu design [45] can be considered with the larger concept of “nudging” where the choice architecture is designed to facilitate healthier and more sustainable choices.

Although not unexpected, a main observation from this review is that one size does not fit all when it comes to evaluation of food choice and nutrition research. Indicators for outcome measures vary and include nutrition knowledge [28,42], healthy food choices [24,30,38,40,41], food purchases [33,45] or purchase intentions towards healthier foods [29], self-reported intake of fresh produce [28], intake of fruits and vegetables [25,40] or food an nutrient intake [27].

Additionally, and not unexpectedly, this review of reviews highlights that the field of food choice and nutrition is multidisciplinary and applies multiple methodologies. Qualitative research or mixed methods papers provided deeper insights into determinants of food choices [25,26,29,34,35,42], while quantitative papers provided more measurable effect sizes [24,40,41,44] useful, e.g., for future sample size calculations. Systematic reviews are not yet a cross-disciplinary accepted methodology as the authors of the reviews come mainly from health/nutrition and life science arenas [22,26,30,32,33,39,40,41]. It is underscored that some of the reviews are by authors from business economics [38], food science [36], sensory science [31] and marketing [29], highlighting the potential for cross-disciplinary collaboration.

## 4. Discussion

Although the liking of a given food item is the main determinant of being chosen by consumers [51], only one review focused on sensory modalities as explanation of food choice [31]. Moreover, taste and hedonic experience is crucial to shaping food choices during the different periods of life, although the mechanisms might be different [52]. Preconceptions or expectancy about taste is a main barrier towards the adoption of healthier plant-based foods in high meat consumption societies [53], nevertheless, in the review by Munt [34] taste was reported either among the barriers or enablers of healthy food choices. Additionally, the search for sensorial and intellectual pleasure will have an effect on the actual food choices [54]. Learning to obtain pleasure from eating is a process, as well as an opportunity to shape healthy eating behaviours from early childhood [55,56].

Several papers advocated for combining strategies when addressing food choices and their relation to nutrition [20,21,38]. Interventions combining nudges, education, vegetables provision, plant-based recipes may be effective in empowering more vulnerable people to make healthier and more sustainable choices [28], overcoming the social gradients in nutritional inequality [57]. Lifestyle modification, including exercise and a healthier diet (low-fat diet rich in complex carbohydrates fresh fruits, vegetables) are associated with the reduction in the incidence of Type 2 Diabetes mellitus and improved insulin sensitivity [58]. Energy density and portion size are dietary variables that influence behaviour [59], hence, additional professional support by dietitians [60] can improve glycaemic control, decreases the risk of long-term complications and support in weight management.

Responding to the encouragement for a comprehensive approach to food choice and nutrition, new tools are now available and will have to be used in future research. In particular, the Precision Nutrition Approach integrates different kinds of “big data” to reveal the complexity and diversity of human metabolism in response to diet. The tools include genomics, metabolomics, microbiomics, phenotyping, high-throughput analytical chemistry techniques, longitudinal tracking with body sensors, informatics, data science, and sophisticated educational and behavioural interventions [61].

The strategies for addressing child eating behaviours reported earlier are in agreement with a review by Scaglioni et al. [62] that highlighted the effectiveness of covert control, avoidance of food rewards, promoting self-regulation, a more authoritative parenting style, family meals, and highlighted ways in which parents can contribute to making the family environment [21,39] conducive to a healthier lifestyle for the child. Eating meals with the family and thus more traditional food consumption is part of an overall support system that allows children to make a better selection of foods (fewer fast-foods and snacks) [26,47], as well as to maintain a healthier weight status [22].

Strategies to address the growing segment of older consumers should consider comprehensive interventions for meal provision and sustained health [36] taking into account the fact that modern older consumers value socializing and independent cooking [63]. Additionally, designing and providing meals for older consumers can take into account flavour and texture modifications compensating for losses in masticatory and chemosensory ability and thus, enhancing the appreciation of foods and stimulating food intake, especially among the less dependent elderly with poorer health [64].

Food choices at the point of consumption or purchase are the result of many cues that “nudge” people and make their choices easier [38] (although not necessarily healthier or more sustainable). Behavioural interventions can contribute to weight management [43] and weight management success may be larger if the focus is on the consumption of healthy foods of plant origin [12]. In foodservice operations, the design of the buffet can facilitate the intake of foods of plant origin if they are placed at the beginning of the line [65] or when consumers are allowed to self-compose their salads [66]. Changing default policies [67] are advocated by Weihrauch-Blüher [20] and effective in behavioural laboratory settings [66,68]. The implementation of nudges in real-life operations is still inconsistent. On the one hand, operationalizing among adolescents and older consumers in four EU foodservice settings the “dish-of-the-day” was unsuccessful to promote an innovative plant-based alternative to meat [69,70,71] but it was more effective in a restaurant setting and when the choices made were between unfamiliar dishes [72]. In retail settings, customers’ implicit beliefs about the relationship between taste and healthfulness, bringing reusable bags to the store, making multiple choices in a row, receiving real-time feedback on spending while on a budget, and paying with a credit/debit card are all linked to less healthy choices [29].

There is growing evidence of the role of online tools as contributors to healthier food choices [23,25]. A positive impact on vulnerable mothers includes the provision of culturally acceptable recipes and trustworthy information online [73].

This paper has, of course, strengths and limitations. PRISMA guidelines [17,74] and a set of pre-determined inclusion and exclusion criteria were used to have an objective procedure for inclusion and data extraction of data. This umbrella review synthetized 26 systematic reviews. The main limitation is that many review papers [58,62,75,76], essays [54] or scientific opinions [59,77] were not kept for additional data extraction, and many of their findings were omitted from this review or kept for discussion. Although most scientific communications have an abstract in the English language, relevant systematic reviews written in other languages could have been omitted as the search only included documents in English. Additionally, this paper was written by one single author, which will always risk reflexivity bias. It is the belief that the strict methodology and the clear description can make this umbrella review reproducible by other researchers and that the papers obtained with the described procedure will be the same.

## 5. Conclusions

Indicators for outcome measures on food choice and nutrition studies vary widely, and include among others, nutrition knowledge, healthy food choices, food purchases or purchase intentions towards healthier foods, self-reported intake of fresh produce, intake of fruits and vegetables or food and nutrient intake. A common measure is hard to envisage; therefore, multidisciplinary understanding is advocated. Systematic reviews are being used beyond the medical sciences and will be welcome in cross-disciplinary fields such as food choice and nutrition.

The most common strategy implemented to alter food choice with a nutritional aim is nutrition education, followed by the provision of information through labels. Strategies directed towards achieving healthy food choices among children would be more successful if parents are involved. In general, combining strategies seems to be the most effective way to achieve healthier food consumption (e.g., more foods of plant origin) and to maintain a good nutritional status and intake in all age groups.

## Figures and Tables

**Table 1:** Characteristics of the reviews included.

Population Group	First Author	Yr	Country Where the Review Was Performed	Review Design	Number of Articles Included	Intervention Focus	Reported Outcomes
**Infants**	Weihrauch-Blüher [20]	2018	Germany	Systematic Review	95 RCT	Obesity Prevention measures, breastfeeding promotion (exclusive first 4–6 mo), awareness & knowledge transfer to parents, caregivers and nurseries	Infant and pre-school child recommendations: infants exclusive breastfeeding 4–6 mo, include a varied diet with ample beverages (water & unsweetened/sugar-free drinks), ample plant-based foods (vegetables, fruits, whole grain products, potatoes), limited foods of animal origin (milk, milk products, meat, fish, eggs), and a low consumption of sugar and sweets.
**Children**	Matwiejczyk [21]	2018	Australia	Umbrella Review (Systematic review of reviews)	14	Delivery of interventions (staff group education and training sessions, written materials, the inclusion of nutrition-related activities in the childcare curriculum and food and nutrition policies.; Educators as role models influence children acceptance. Interactive education activities as part of the curriculum and using other children as role models. Any involvement from parents is associated with positive outcomes	Interventions to promote healthy eating in children aged 2–5 years attending centre-based childcare are effective; Successful interventions were multi-component, multi-level targeting both environmental and individual-level determinants of healthy eating behaviours. Multi-component interventions included educational strategies, changes to the centre-environment and policy. Interventions based on Social Cognitive Theory and Social Learning Theory yielded significant favourable outcomes.
	Weihrauch-Blüher [20]	2018	Germany	Systematic Review	95 RCT	Obesity Prevention measures Schools (children and adolescents): Provision of physical activities; implementation of sugar/fat taxes; binding standards for the catering offers; ban the advertisement of unhealthy foods targeted at children.	Children in school age, same as for infants (see above): but special emphasis on avoiding or limit sugary beverages.
	Young [22]	2018	Australia	Systematic Review	16	Presence of grandparents at home	The odds of being obese was about 1.5 (OR range 1.47–1.72) in Japan, China and the USA; and 4 times in Greece when grandparents prepared meals. This was not the case with Hispanic children in the USA: grandparents at home were associated with lower BMIz-scores.
**Adolescents**	Chau [23]	2019	USA	Systematic Review	19	Social media as component of nutritional interventions	11 out of 16 Interventions for adolescents and young adults that included social media showed short-term positive nutrition-related clinical or behavioral outcomes.
	Christoph [24]	2019	USA	Systematic Review and Meta-Analysis	21	Calorie labelling	Nutrition labels were found to have a moderate but significant positive effect on dietary choices in college students. Controlled studies showed contradictory results. Pre-post interventions showed a weighted mean reduction of 36 calories. Contextual labels (traffic lights or daily recommended intake) had higher efficacy.
	Hsu [25]	2019	Australia	Systematic Review	14	Different behaviour change techniques (e.g., Goal setting, social support, self-monitoring, behavioural contract, social comparisons, problem solving, demonstration “how-to-do”, etc.)	Fruit & vegetables consumption was the most successfully targeted behaviour, with significant improvements. Sugar-sweetened beverages reduction was also achieved, but no impact reported on fast foods and highly processed foods.
	Wrottesley [26]	2019	South Africa	Systematic Review	67	Rural adolescents who maintained more traditional eating behaviours than their urban counterparts; urban adolescents pronounced change with age;	Rural adolescents, more likely to partake in family meals and to consume fewer fast-food and snack-food item; urban adolescents, lower fruit, vegetable and dairy intakes and higher processed meat, oil, fast-food and sugar-sweetened beverage intakes reported at older ages
	Weihrauch-Blüher [20]	2018	Germany	Systematic Review	95 RCT	Obesity Prevention measures Schools (children and adolescents): Provision of physical activities 90 min/day; multicomponent approach.	Adolescents: Positive weight and body composition (waist circumference, body fat mass) effects observed when a multi-component approach was used with an interdisciplinary intervention concept using direct transfer of knowledge to the adolescents themselves
	Noll [27]	2017	Brasil	Systematic Review	21	Sport training and modalities; sociodemographic differences; meal patterns; menu style; having a nutrition plan	Food and nutrient intake. Athletes do not modify their eating patterns to the demands of training. Mostly information on nutrients but not on actual foods eaten.
**Adults**	An [28]	2019	USA	Systematic Review	14	Nutrition education interventions and the client-choice intervention	Enhanced participants’ nutrition knowledge, cooking skills, food security status and fresh produce intake
	Castro [29]	2018	USA	Scoping Review, applying systematic procedure	41	Shelf display and product factors (branding, nutrition labelling, food sampling); pricing and price promotion factors, that work during the intervention but are not permanent; and in-store and customer decision-making factors (e.g., Immediate feedback helps people on a budget)	Purchase intentions and choice of healthier foods
	Chau [23]	2019	USA	Systematic Review	19	Social media as component of nutritional interventions	11 out of 16 Interventions for adolescents and young adults that included social media showed short-term positive nutrition-related clinical or behavioural outcomes.
	Christoph [24]	2019	USA	Systematic Review and Meta-Analysis	21	Calorie labelling	Nutrition labels were found to have a moderate but significant positive effect on dietary choices in college students. Controlled studies showed contradictory results. Pre-post interventions showed a weighted mean reduction of 36 calories. Contextual labels (traffic lights or daily recommended intake) had higher efficacy.
	Powell [30]	2019	Australia	Scoping Review, using systematic approach	99	Physical and social contexts associated to food choices made by 19- to 24-y-old young adults in the USA.	Food choices in following categories: SSBs (including energy drinks and coffee); fruits and vegetables (about 2 servings/day); International foods (ethnic/global inspire foodservice and home consumption); convenience foods (mess-free, portable for on the go; entrees for reheating at home); snack foods (mini-meals; cheap meals substitutes); healthy foods (better-for-you); customizable foods (selecting individual components at point of purchase); foods from sustainable production methods (organic, non-GMO, updated familiar dishes with healthier ingredients); interesting foods (sense of adventure in food, mostly limited time menu options); Regional foods (long held regional traditions influence food preparation and adequate foods for occasions).
	Tan [31]	2019	Australia	Systematic Review	17	Taste sensitivity (thresholds), intensity, or hedonic responses to sweet stimuli	Food intake. Hedonic measurements were more likely to be associated with dietary intake, but the results were inconsistent through the 17 revised papers.
	Verghese [32]	2019	USA	Scoping Review, using systematic approach	16	(1) monetary incentives (2) nutrition education, and (3) combined nutrition education plus monetary incentives.	Monetary interventions showed modest improvements in reported fruit and vegetable intake among SNAP beneficiaries. Nutrition education interventions showed improvement in psychosocial correlates of diet, changes in dietary intake were inconsistent. Combination programs demonstrated the strongest improvements in dietary change among beneficiaries.
	Kaur [33]	2017	UK	Systematic Review and Meta-Analysis	31 papers; 17 in meta-analysis	Health Claims on Food Labels	Actual food purchases, consumption or stated intention. The meta-analyses of 17 studies found that health-related claims increase consumption and/or purchasing (OR 1.75, CI 1.60–1.91).
	Munt [34]	2017	Australia	Scoping Review using systematic approach	34	The comprehensive and complex factors that contribute to dietary behaviours and subsequently in weight management amongst young adults. Identification of barriers and enablers of healthy eating.	Barriers towards healthy eating: male apathy towards diet; unhealthy diet of friends and family; expected consumption of unhealthy foods in certain situations; relative low cost of unhealthy foods; lack of time to plan, shop, prepare and cook healthy foods; lack of facilities to prepare, cook and store healthy foods; widespread presence of unhealthy foods; lack of knowledge and skills to plan, shop, prepare and cook healthy foods; lack of motivation to eat healthily (including risk-taking behaviour). The key **enablers** towards healthy eating: female interest in a healthy diet; healthy diet of friends and family; support/encouragement of friends and family to eat healthy; desire for improved health; desire for weight management; desire for improved self-esteem; desire for attractiveness to potential partners and others; possessing autonomous motivation to eat healthy and existence and use of self-regulatory skills.
	Pitt [35]	2017	Australia	Systematic Review	30	The role of build environments and its contribution to diet and health outcomes such as obesity. Identification of barriers to healthy eating. Socio-ecological determinants of food choices. Food shopping.	Theme 1: Community nutrition environment, Availability, accessibility, affordability; Theme 2: Consumer nutrition environment, In-store food availability, food store characteristics/features; Theme 3: Other environmental factors, Influence of media and adverts, other; Theme 4: Individual coping strategies within the community nutrition environment & within the consumer nutrition environment
	Zhou [36]	2018	Denmark	Systematic Review	16	Interventions consisting of: Dietary education, Meal service provision, Multi-components	Nutrition education has modest effect on dietary change. Meals interventions improve nutritional status. Comprehensive interventions combining nutrition education and provision of healthy foods can improve diet quality.
**Pregnant women**	Kavle [37]	2018	USA	Systematic Review	23	Identification of the role that cultural beliefs and food choices have on adequate nutrition during pregnancy. Identification of the main drivers of food choice in this group.	Barriers to adequate nutrition during pregnancy included cultural beliefs related to knowledge of quantity of food to eat during pregnancy, amount of weight to gain during pregnancy, and “eating down” during pregnancy for fear of delivering a large baby. Foods considered inappropriate for consumption during pregnancy or lactation contributed to food restriction. Drivers of food choice were influenced by food aversions, economic constraints, and household food availability.
**General, in more than one age group, or not specified**	Bauer [38]	2019	Denmark	Systematic Review	39	Several nudging strategies: improving the provision of nutritional information; Nutritional Information in Supermarkets and on Pre-packaged Foods; Nutritional Information in Restaurants and on Menus; Making Health Salient and Healthy Food Choices the Norm; Priming; Social Norms; Using Healthy Defaults, positioning, Presentation; Portion Size; Food variety; Incentivize Healthier Choices and Pre-planning of Food Choice	15% more healthy choices; modestly significant and positive effect of nudging interventions altering placement and properties of food choice, sales, and servings (Cohen’s *d* = 0.3). Menu labels reduce 78–100 kcal; health claims increase 75% healthy choices;
	Bhana [39]	2018	New Zealand	Systematic Review	26	Attitudes, knowledge, use of labels, sociodemographic characteristics	Salt consumption: Strategies for reduction are “self-control at home/table”, use of herbs/spices, avoidance of processed foods, pre-packaged meals, fast food restaurants and requesting low/no salt options. Also purchasing foods with labels “low/reduced o no salt/sodium”
	Hosseini-Esfahani [40]	2018	Iran	Systematic Review on the publications of the Teheran Study	105	Adherence to healthy food choices	Higher adherence to healthy food choices was associated with reduced odds of MetS, abdominal obesity, dyslipidaemia and hypertension.
	Hosseini-Esfahani [41]	2018	Iran	Systematic Review on the publications of the Teheran Study	52	Adherence to healthy food choices.	Odds of chronic kidney disease 2-fold by sugar sweetened beverage; 2,5-fold by sugar sweetened carbonated soft drinks. Higher adherence to healthy food choices was associated with reduced odds of dysglycemia and CVD. Dietary sources of renal-protective nutrients should be encouraged among the general population.
	Perry [42]	2017	Canada	Scoping Review with systematic approach	19 articles, 30 grey literature	The impact of food literacy on healthy diets and to evaluate the outcomes of food literacy interventions.	1. Food and Nutrition Knowledge informs decisions about intake and distinguishing between ‘healthy’ and ‘unhealthy’ foods. 2. Food Skills focuses on techniques of food purchasing, preparation, handling and storage. 3. Self-Efficacy and Confidence represent one’s capacity to perform successfully in specific situations. 4. Ecologic refers to beyond self and the interaction of macro- and microsystems with food decisions and behaviours. 5. Food Decisions reflects the application of knowledge, information and skills to make food choices.
	Rolls [43]	2017	USA	Review with systematic search	10	high vs. low energy density foods	Energy density influences intake through a complex interplay of cognitive, sensory, gastrointestinal, hormonal and neural influences. Lower density foods in meals can help with satiety and reduction of overall energy intake while improving the quality of the diet.
	Seyedhamzeh [44]	2018	Iran	Systematic Review and Meta-Analysis	8	Calorie labelling	No significant effect on the amount of kcal chosen or on healthy food choices
	Sacco [45]	2017	Canada	Systematic Review	11	Menu labelling in artificial and real-world settings. Menus displaying numeric calorie information; calories plus a contextual statement on average daily caloric requirements for adults; calorie content with additional nutrition information, such as fat content or Nutrition Facts label; interpretative information to denote a ‘healthier choice’ (e.g., heart or apple); nutrition bargain price; traffic light system plus a legend describing the meaning of the colour ratings; calories alongside physical activity equivalents.	Quantity of calories purchased in foodservice: Lab situation: Parents: 100–200 kcal reduction when numeric calorie contents was displayed next to the menu; Children: 158 kcal reduction by ‘healthy choice symbol’ with a contextual statement or 171 kcal reducing with numeric calorie and fat information; Natural experiments: No effect

**Abbreviations in the table:** BMI—Body Mass Index; CVD—Cardiovascular Disease; MetS—Metabolic Syndrome; OR—Odds Ratio; RCT—Randomized Controlled Trial; SSB—Sugar Sweetened Beverages; SNAP—Supplemental Nutrition Assistance Program.

**Table 2:** Synthesis of interventions to promote healthy eating by age group.

Age Group	Interventions to Promote Healthy Food Choices
Infants	Breastfeeding promotion and knowledge sharing with caregivers and personnel in nurseries
Children	Involvement of parents, role models and binding standards for public catering offers
Adolescents	Social media components, provision of information (labelling), obesity prevention measures including 90 min/day physical activity, behaviour change techniques, healthy eating policies
Adults	Nutrition education (knowledge provision), tasty foods, financial incentives
General across life-stages	Promotion of adherence to healthy food choices (plant-based), changes in the choice environment (nudging), improving food literacy, provision of healthier foods, social media components
